# Are there interindividual differences in the reactive hypoglycaemia response to breakfast? A replicate crossover trial

**DOI:** 10.1007/s00394-024-03467-y

**Published:** 2024-09-04

**Authors:** Javier T. Gonzalez, Lorenzo Lolli, Rachel C. Veasey, Penny L. S. Rumbold, James A. Betts, Greg Atkinson, Emma J. Stevenson

**Affiliations:** 1 Centre for Nutrition, Exercise and Metabolism, University of Bath, Bath, UK; 2 Department for Health, University of Bath, Bath, BA2 7AY UK; 3 Department of Sport and Exercise Sciences, Institute of Sport, Manchester Metropolitan University, Manchester, UK; 4 Department of Sport, Exercise and Rehabilitation, Faculty of Health and Life Sciences, Northumbria University, Newcastle Upon Tyne, UK; 5 School of Sport and Exercise Sciences, Liverpool John Moores University, Liverpool, UK; 6 Faculty of Medical Sciences, Human Nutrition Research Centre, Population Health Sciences Institute, Newcastle University, Newcastle upon Tyne, UK

**Keywords:** Breakfast, Response heterogeneity, Glucose, Metabolism, Carbohydrate

## Abstract

**Background:**

Following consumption of a meal, circulating glucose concentrations can rise and then fall briefly below the basal/fasting concentrations. This phenomenon is known as reactive hypoglycaemia but to date no researcher has explored potential inter-individual differences in response to meal consumption.

**Objective:**

We conducted a secondary analysis of existing data to examine inter-individual variability of reactive hypoglycaemia in response to breakfast consumption.

**Methods:**

Using a replicate crossover design, 12 healthy, physically active men (age: 18–30 y, body mass index: 22.1 to 28.0 kg⋅m^− 2^) completed two identical control (continued overnight fasting) and two breakfast (444 kcal; 60% carbohydrate, 17% protein, 23% fat) conditions in randomised sequences. Blood glucose and lactate concentrations, serum insulin and non-esterified fatty acid concentrations, whole-body energy expenditure, carbohydrate and fat oxidation rates, and appetite ratings were determined before and 2 h after the interventions. Inter-individual differences were explored using Pearson’s product-moment correlations between the first and second replicates of the fasting-adjusted breakfast response. Within-participant covariate-adjusted linear mixed models and a random-effects meta-analytical approach were used to quantify participant-by-condition interactions.

**Results:**

Breakfast consumption lowered 2-h blood glucose by 0.44 mmol/L (95%CI: 0.76 to 0.12 mmol/L) and serum NEFA concentrations, whilst increasing blood lactate and serum insulin concentrations (all *p* < 0.01). Large, positive correlations were observed between the first and second replicates of the fasting-adjusted insulin, lactate, hunger, and satisfaction responses to breakfast consumption (all *r* > 0.5, 90%CI ranged from 0.03 to 0.91). The participant-by-condition interaction response variability (SD) for serum insulin concentration was 11 pmol/L (95%CI: 5 to 16 pmol/L), which was consistent with the τ-statistic from the random-effects meta-analysis (11.7 pmol/L, 95%CI 7.0 to 22.2 pmol/L) whereas effects were unclear for other outcome variables (e.g., τ-statistic value for glucose: 0 mmol/L, 95%CI 0.0 to 0.5 mmol/L).

**Conclusions:**

Despite observing reactive hypoglycaemia at the group level, we were unable to detect any meaningful inter-individual variability of the reactive hypoglycaemia response to breakfast. There was, however, evidence that 2-h insulin responses to breakfast display meaningful inter-individual variability, which may be explained by relative carbohydrate dose ingested and variation in insulin sensitivity of participants.

**Supplementary Information:**

The online version contains supplementary material available at 10.1007/s00394-024-03467-y.

## Introduction

Postprandial metabolic responses to a mixed-macronutrient meal typically include a shift from predominantly fat to predominantly carbohydrate metabolism. This shift is reflected by a transient increase in circulating glucose concentrations, caused largely by appearance of ingested carbohydrate [1]. There is also an increase in systemic insulin concentrations which suppresses endogenous glucose production and adipose tissue lipolysis [2], and stimulates peripheral tissue glucose uptake and glycolysis [2]. These responses contribute to buffering the glucose excursion [3], characterised by increased carbohydrate oxidation rates and circulating lactate concentrations, decreased fat oxidation rates, and a return of glucose concentrations to fasting concentrations [3].

In some scenarios, the recovery of circulating glucose concentrations after ingestion of a meal can “overshoot” and “dip” below their basal/fasting concentration before homeostasis is fully restored [4]. This phenomenon is known as reactive hypoglycaemia and is associated with a variety of symptoms including fatigue, light-headiness, sweating and irritability. It has recently been suggested that reactive hypoglycaemia may also be relevant for appetite control [4]. Small correlations (*r* < 0.3) were observed between a more pronounced reduction in glucose concentrations 2 h after a meal and increased feelings of hunger (*r* = 0.16, *p* < 0.001) and the reporting of greater energy intake over the subsequent 24 h (*r* = 0.27, *p* < 0.001) [4]. Within-individual analyses revealed a negligible (*r* < 0.1) association between the day-to-day variation in postprandial glucose reductions at 2–3 h and the day-to-day variation in 24-h reported energy intake (*r* = 0.06, *p* < 0.001) [4]. Based on this work, it has been suggested that there may be inter-individual differences in the metabolic response to meals with relevance to appetite control, although the small size of the between-variable correlations combined with the free-living conditions under which participants were testing, make inferences regarding the interindividual heterogeneity of each variable of interest somewhat unclear.

Establishing true inter-individual variability of a measured variable in response to an intervention requires repeated administration of experimental conditions or interventions within the same individuals, with randomisation in the order of exposures to control and intervention [5–7]. This approach has been termed a repeated period (*replicate*) randomised crossover design, which is a form of n-of-1 trial [5–7]. *N*-of-1 trials involve repeated administration of the intervention or control within individuals and there is an example of this design in the context of individual response heterogeneity of antihypertensive drugs [8]. Only with repeated administration of treatment and control conditions can the necessary participant-by-treatment interaction be derived (i.e., the variation between people in the effects of a treatment such as diet). The practical/clinical relevance of treatment response heterogeneity is that, when a true and clinically meaningful participant-by-treatment interaction is identified, then this facilitates rational approaches to personalised treatment. In other words, knowledge about appropriately quantified response heterogeneity provides the rationale to treat individual people differently or with a generalised “cover-all” treatment approach. The use of a replicate crossover design within nutrition research is, to date, rare. In one recent report, the researchers examined the glucose, insulin, and appetite responses to a meal with a replicate crossover design, focussing on the initial 30–60-min postprandial period [9]. It was reported that there was evidence for true inter-individual differences in the responses of peak glucose concentrations and hunger ratings in response to a breakfast [9]. However, since blood sampling finished at 60 min after the meal, it is not known whether reactive hypoglycaemia, which typically occurs between 1 and 3 h postprandially [4, 10], displays true interindividual variability in response to a meal. We previously reported on a study examining the interactive effects of breakfast and exercise on mean changes in postprandial metabolism later in the day. The design of this study was, essentially, a replicate crossover design for the initial 2 h of the study day. Therefore, the aim of this study was to examine whether there was evidence for true inter-individual variability in the metabolic response to a breakfast consumption over a 2-h period. We focused on the 2-h change in glucose as the primary outcome to understand whether there is evidence that reactive hypoglycaemia displays inter-individual response variability. We hypothesised that 2-h glucose concentrations display meaningful inter-individual variability in response to breakfast consumption.

## Methods

The original study design was a randomised crossover with 4 conditions, fasted prior to rest, fasted prior to exercise, breakfast prior to rest and breakfast prior to exercise and is available open access [10]. Up to the exercise period (and parallel period in control), these conditions provided, essentially, a replicate crossover with two control and two breakfast conditions. The data collected peri-exercise are not included in the present study. Therefore, the conditions are first fasted visit, second fasted visit, first breakfast visit and second breakfast visit. The methods have been reported in detail previously and are described in brief herein. Participants were 12 young, healthy males who provided written informed consent prior to participation. The protocol was approved by the School of Life Sciences Ethics Committee at Northumbria University, and the study was conducted in line with the latest version of the Declaration of Helsinki.

Participants arrived at the laboratories at 0730 in the overnight fasted (10–14 h), rested state for 4 separate study visits, with the condition sequence randomised for each participant (unrestricted randomisation performed using randomizer.org by J.T.G), with at least a 7-day washout. The study was conducted as open label by necessity of meal consumption. Participants were asked to replicate food intake and physical activity for the day before study visits, and asked to avoid alcohol, caffeine, and vigorous activity for 24 h prior to study visits.

Upon arrival at the laboratory, a cannula was inserted into an antecubital vein for repeated blood sampling. A baseline blood sample was taken, alongside a 5-minute measurement of expired gases via indirect calorimetry (Metalyzer 3B, Cortex). Visual analogue scales (VAS, 0–100 mm scale) were then completed to assess ratings of hunger, fullness, satisfaction, and prospective consumption Participants then ingested a test breakfast or remained fasted (water was permitted *ad libitum*). Further 5-minute breath samples were taken every 30 min after breakfast consumption, and a further blood sample and set of VAS were taken 2 h after breakfast consumption. Participants were familiarised with the visual analogue scales on a preliminary visit prior to the main study days.

### Test breakfast

The breakfast comprised 72 g instant oats (Oatso Simple Golden Syrup, Quaker Oats, PepsiCo, UK) and 360 mL semi-skimmed milk (Tesco, UK) which provided 1859 kJ (444 kcal) energy, 67 g carbohydrate, 19 g protein and 11 g fat.

### Blood sampling and analysis

10-mL blood samples were collected whilst participants were seated upright to control for postural changes to plasma volume. From the 10-mL blood samples, a 20-µL capillary tube was filled with whole blood to determine blood glucose and lactate concentrations (Biosen C_Line, EKF Diagnostics), and 5 mL allowed to stand for 30 min in a non-anticoagulant tube before being centrifuged at 3000 rpm at 4 °C for 10 min. Aliquots of serum were then stored for later determination of NEFA (WAKO Diagnostics) and insulin (DIAsource ImmunoAssays S.A.) concentrations in duplicate. All serum samples were stored at − 80 °C. The intra-assay CV were 5.6 and 7.2% for NEFA and insulin, respectively. Inter-assay CV were 8.1 and 3.6 for NEFA and insulin, respectively. To reduce the inter-assay variation, samples from each participant were analysed during the same run where possible.

### Breath sampling and analysis

Expired gas samples were collected using an online gas analysis system (Metalyzer 3B, Cortex) calibrated using gases of known concentrations and a 3-L syringe. Participants wore a facemask and after a 2-min stabilisation phase, 5-min samples were obtained and averaged. Carbohydrate and fat oxidation rates were calculated assuming negligible protein oxidation, using stoichiometric equations [11]. Since the baseline sampling was not in accordance with best practice measures for resting metabolic rate [12] and could thereby introduce additional error into the postprandial change measures, the data for energy expenditure, carbohydrate oxidation and fat oxidation are not adjusted for baseline.

### Statistical analysis

Although the sample size for the present study has been set a priori by the previous study (for detection of mean treatment effects), we can estimate minimal detectable effect sizes that are relevant to response heterogeneity for our sample size of 12 participants. In terms of the between-replicate correlation coefficient (see below), 12 participants would translate to a between-replicate correlation of 0.5 as being statistically significant (one-tailed *P* = 0.049). The 90% confidence interval for this correlation would be 0.00 to 0.80. A directional (one-tailed) hypothesis is relevant in this context because both a zero and a negative correlation would mean non-rejection of the null hypothesis (*r* ≤ 0). Adjustment for multiple comparisons due to secondary outcomes was not undertaken on the basis that if secondary outcomes are interpreted precisely and exclusively, then the per-comparison-wise error rate is not increased [13].

The analysis framework in the present study involved a four-step approach consistent with previous research adopting similar designs and methodological standards for the analysis of replicate crossover trials [9, 14–16]:

1. The association between the first and second replicate of the control-adjusted individual treatment effects was quantified for each outcome using Pearson’s product-moment correlation coefficients. Pearson’s correlation was selected over an intraclass correlation coefficient because the latter statistic traditionally pools systematic bias (in our case, the mean treatment effect) and random individual variability together, which would mask appropriate quantification of the individual consistency in response. The first breakfast condition in any participant’s sequence was paired to the first control condition in the same individual’s sequence. Thresholds of 0.1, 0.3 and 0.5 were used to infer correlations as small, moderate, and large, respectively [17]. This correlation coefficient quantifies the consistency of the breakfast effect across the replicated experimental conditions.
2. The SD of the of the breakfast condition was adjusted for the SD of the control condition to provide an overall estimate of the true between-participant differences in treatment response using the following equation:

1 $$\:{\text{SD}}_{\text{IR}}\text{=}\sqrt{{\text{SD}}_{\text{Breakfast}}^{\text{2}}\text{-}{\text{SD}}_{\text{Control}}^{\text{2}}}$$

Where SD_IR_ represents the true interindividual variation in treatment effect. SD_Breakfast_ and SD_Control_ are the SDs of the pre-to-post change scores for the breakfast and fasting control conditions (averaged over the 2 replicates using the relevant equation for pooling SDs [18]. This SD is a naïve estimation of individual response variability adjusted for any random trial-to-trial variability (quantified using the comparator condition data).

3) Whilst Eq. 1 estimates response variance adjusted for the change variance in the control condition, the associated SEs and CIs are not appropriate for a within-participant crossover study design. Therefore, we also performed a within-participant linear mixed model. Using the MIXED procedure in SAS OnDemand for Academics (SAS Institute), a within-participant linear mixed model was formulated to quantify participant-by-condition interaction for each outcome [19]. Each included condition and period (sequence), and their interaction, were modelled as fixed effects with participant plus participant-by-condition interaction term modelled as random effects (refer to the SAS code supplied in Supplemental Methods). Standard residual diagnostics were undertaken to assess the “influence diagnostics” of a potential set of observations on the adequacy and the stability of the modelled covariance parameter estimates [20–22]. These analyses were also performed for circulating metabolite and insulin concentrations with and without baseline adjustment to explore the effect of baseline adjustment in analyses of response heterogeneity.
4) Using the approach recently reported by Senn [16], we calculated the replicate-averaged treatment effect for each participant along with the respective 95% confidence intervals (CI). A sample estimate of within-subjects variance is calculated and converted to a standard error using appropriate degrees of freedom given the completed cycles to derive per participant replicate-averaged treatment effects [16]. A random-effects meta-analysis approach summarised each individual-participant replicate-averaged treatment effect and respective standard errors of the mean effect (SE) using the *metagen()* function available in the *meta* package [16, 23]. The tau-statistic (τ) value described the between-participant variability across the distribution of true replicate-averaged treatment effects [24, 25]. The restricted maximum-likelihood estimation method determined the mean τ-statistic value with uncertainty surrounding the point estimate described as 95%CI derived using the generalised Q-statistic method [26]. Weighted raw mean replicate-averaged treatment effect differences were reported as descriptive statistics with the 95% prediction interval (95% PI) describing the expected range for the distribution of true mean differences for 95% of similar future studies [27, 28]. Meta-analyses were conducted in R (version 3.6.3, R Foundation for Statistical Computing).

Existing literature and information in this field informed minimal clinically important differences (MCID; ∆) definitions [29]. A target difference in glucose concentrations of ∆=±0.5 mmol/L on the basis that this is the smallest difference shown to be associated with mortality [30]. A target difference in insulin concentrations of ∆=±6 pmol/L as the smallest difference shown to have a meaningful effect on non-esterified fatty acid (NEFA) turnover rates [2]. A target difference in NEFA concentrations of ∆=±0.29 mmol/L as the smallest difference shown to affect skeletal muscle insulin signalling [31]. A target difference in lactate concentrations of ∆=±0.5 mmol/L on the basis that this is the typical rise seen after a mixed-macronutrient meal [32], and is also the target difference used as a threshold in exercise testing [33]. For appetite ratings a ∆=±5 mm was selected as the target difference as suggested by authors in the field [34].

Pearson’s product-moment correlation coefficients were quantified between the pooled mean control-adjusted meal response for the change in glucose and the pooled mean control-adjusted response for all other outcomes to assess whether reactive hypoglycaemia was associated with responses of other metabolites or hormones, or appetite ratings. Finally, to explore whether participant characteristics were associated with postprandial responses, Pearson’s product-moment correlation coefficients were quantified between the participant age, height, body mass and body mass index, and the pooled mean control-adjusted response for all other outcomes.

## Results

### Participant characteristics

Participant characteristics have been reported previously and are reproduced in Table 1.

**Table 1:** Participant characteristics

	Total sample (*n* = 12)	Sample with blood data (*n* = 11)
Age (y)	23 ± 4	23 ± 4
Height (m)	1.78 ± 0.07	1.79 ± 0.07
Body mass (kg)	77.2 ± 5.3	77.1 ± 5.5
Body mass index (kg⋅m^− 2^)	24.5 ± 2.0	24.2 ± 1.7

Data are mean ± SD, *n* = 12 young healthy men

### Circulating metabolites and insulin

**Fig. 1:**
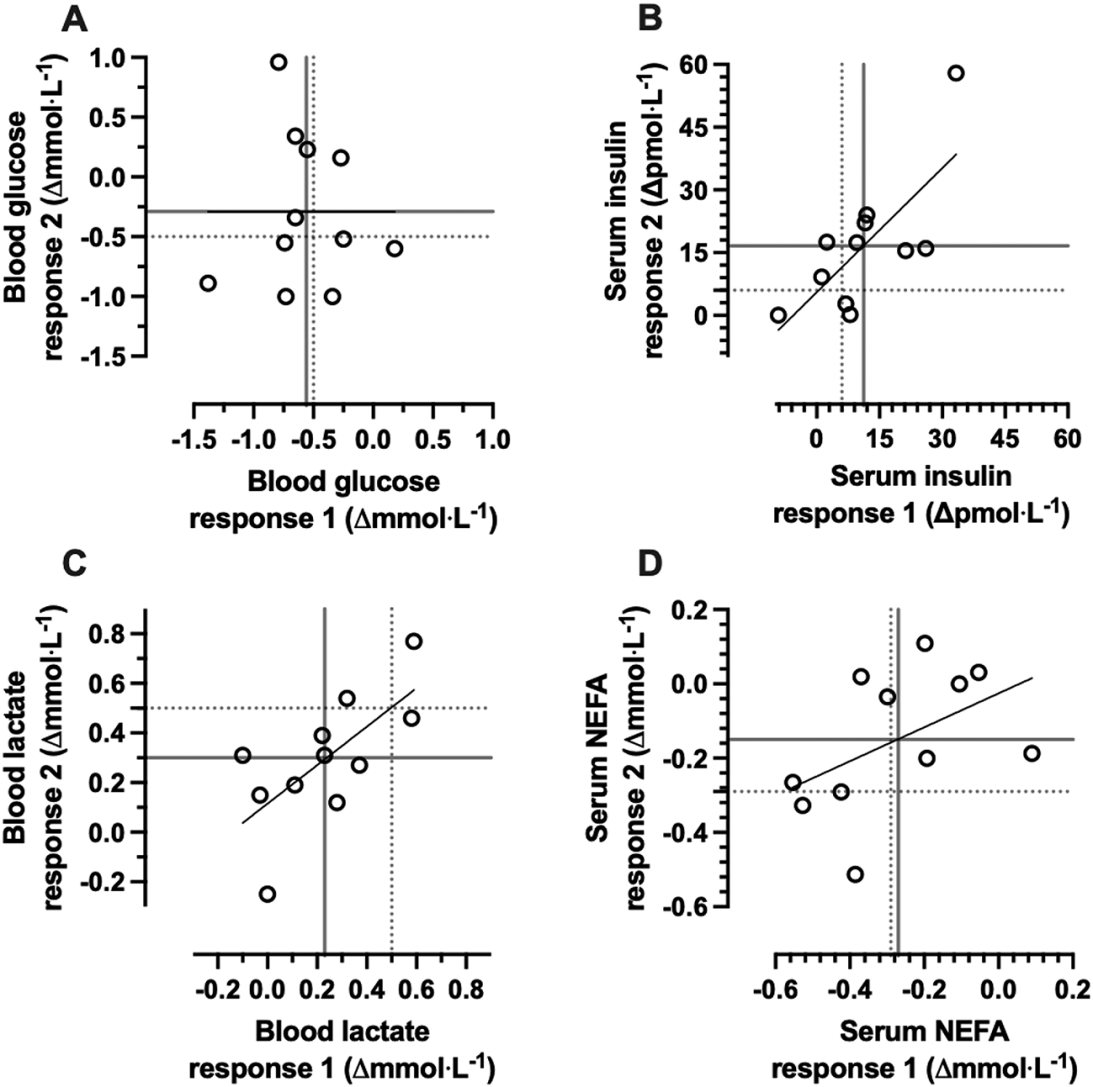
Correlation between the replicates of the baseline-to-two-h response to breakfast minus the fasting control condition, for blood glucose (**A**), serum insulin (**B**), blood lactate (**C**), and serum NEFA concentrations (**D**). “Response 1” corresponds to the first pair of conditions (breakfast 1 minus control 1) and “Response 2” to the second pair of conditions (breakfast 2 minus control 2). Each data point is an individual participant. The dotted lines represent the MCID and the solid lines represent the group mean. *n* = 11. MCID, minimal clinically important difference. NEFA, non-esterified fatty acid

**Fig. 2:**
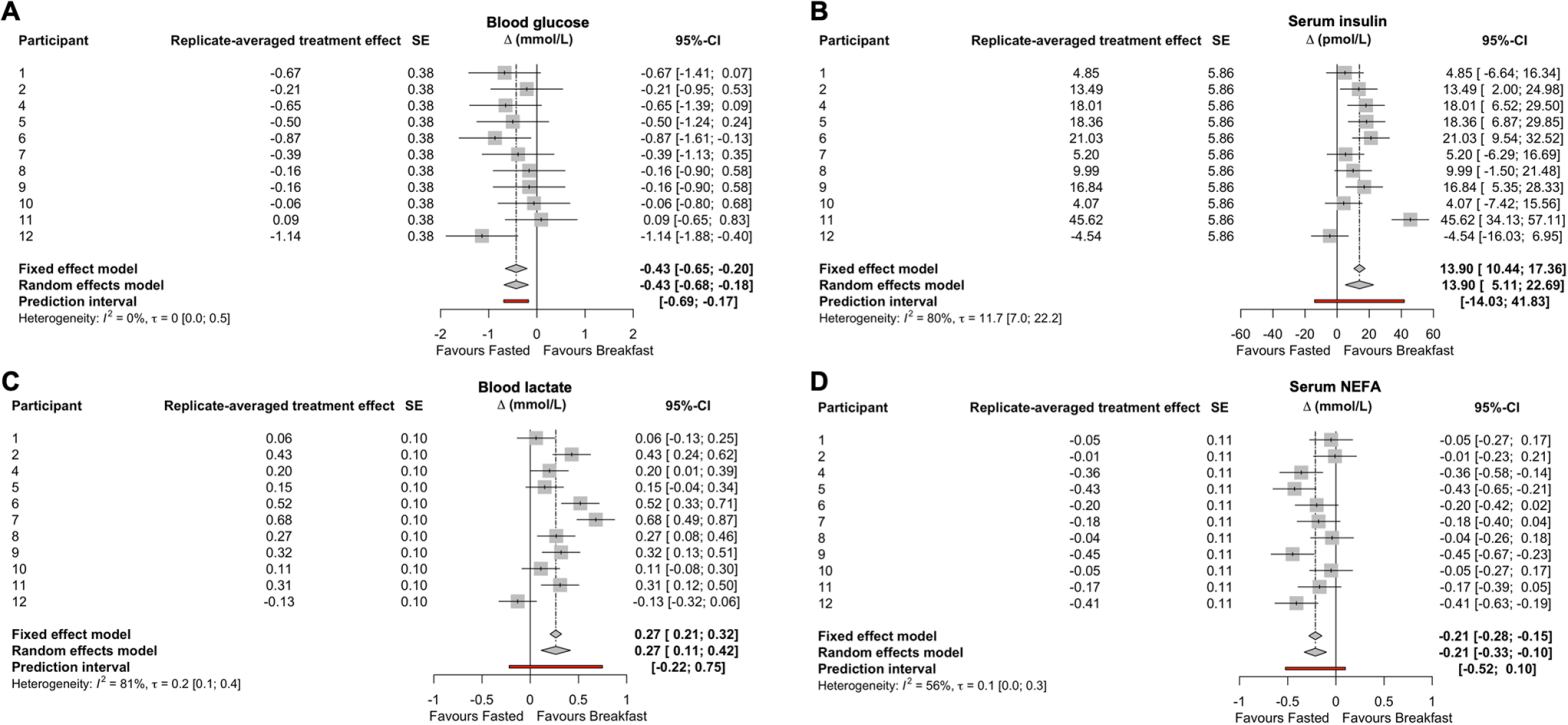
Results of the meta-analysis of each participants treatment effect estimate for blood glucose (**A**), serum insulin (**B**), blood lactate (**C**), and serum NEFA concentrations (**D**) two h after consumption of breakfast (BREAKFAST) relative to two h after remaining in the overnight fasted state (FASTED). *n* = 11. NEFA, non-esterified fatty acid

When taking the average of both breakfast trials and both fasting trials, there was evidence of reactive hypoglycaemia at the group level, as the 2-h change in glucose concentrations demonstrated a greater decline following breakfast than with compared with continued overnight fasting *p* < 0.01, Table 2). A negligible correlation was observed between the 2 replicates of fasting-adjusted 2-h postprandial glucose responses (*r* < -0.001, 90%CI: -0.52 to 0.52, *p* = 0.50, Fig. 1A). Both estimates of the individual differences SD were below the MCID of ± 0.50 mmol/L and the *p* value for the participant-by-condition interaction was above the threshold for statistical significance (Table 2; Fig. 2A). Analysis without adjustment for baseline values did not change this inference (participant-by-condition interaction, *p* = 0.35).

**Table 2:** Estimated marginal means and SEs for primary outcome measures in the fasting and breakfast conditions with the true individual differences SD

Outcome	Mean (SE)			Main effect of condition		Estimate 1^1^		Estimate 2^2^	
	Fasting	Breakfast		Mean difference (95% CI)		Individual differences SD		Individual differences SD (95% CI)	*P* value (int)
Change in glucose									
(mmol/L)	-0.21 (0.06)	-0.65 (0.13)		-0.44 (-0.76 to -0.12)		0.38		0.37 (-0.17 to 0.56)	0.101
Change in insulin									
(pmol/L)	-2.44 (0.72)	11.84 (3.55)		14.28 (5.90 to 22.65)		12.80		11.47 (4.76 to 15.51)	0.018
Change in lactate									
(mmol/L)	-0.01 (0.05)	0.25 (0.06)		0.26 (0.13 to 0.38)		0.16		0.11 (-0.12 to 0.20)	0.394
Change in NEFA									
(mmol/L)	-0.08 (0.03)	-0.32 (0.03)		-0.25 (-0.33 to -0.16)		0.13		0.04 (-0.10 to 0.11)	0.777
Energy expenditure									
(kcal/2 h)	202 [11]	225 [12]		23 (7 to 40)		30		14 (-20 to 28)	0.527
Fat oxidation									
(g/2 h)	16.2 (1.6)	13.1 (1.6)		-3.1 (-4.8 to -1.3)		1.6		-1.1 (-3.1 to 2.6)	0.748
CHO oxidation									
(g/2 h)	15.1 (2.0)	25 (2.0)		10.3 (5.7 to 15.0)		1.6		1.1 (-6.4 to 6.6)	0.952
Change in hunger									
(mm)	10.9 (2.1)	-12.0 (5.0)		-23 (-35.1 to -10.8)		19.5		15.7 (-7.2 to 23.4)	0.106
Change in fullness									
(mm)	-12.5 (2.0)	20.8 (4.5)		33.3 (22.1 to 44.5)		15.5		13.9 (-7.4 to 21.0)	0.127
Change in satisfaction									
(mm)	-13.3 (1.4)	16.0 (2.8)		29.3 (22.2 to 36.4)		15.7		8.4 (-3.7 to 12.4)	0.103
Change in consumption									
(mm)	9.9 (3.4)	-12.8 (3.8)		-22.6 (-31.1 to 14.1)		13.9		5.9 (-10.2 to 13.2)	0.622

Data are presented for *n* = 12 young, healthy men for all variables other than glucose, insulin, lactate and NEFA, for which *n* = 11. NEFA, non-esterified fatty acid. CHO, carbohydrate

^1^Naïve estimation of individual response variability based on Equation 1

^2^2Estimation of individual responsevariability based on a within-participant Linear Mixed Model

In contrast to glucose, the group mean 2-h change in insulin concentrations was higher with breakfast compared with fasting (*p* < 0.01, Table 2). A large positive correlation of 0.74 (90%CI: 0.35 to 0.91, *p* < 0.01, Fig. 1B) was observed between the 2 replicates of fasting-adjusted 2-h postprandial insulin responses. The within-trial SD for insulin was substantially greater during the breakfast trial than the fasting trial, with both estimates of the individual differences SD approximately 2-fold larger than the MCID of ± 6 pmol/L plus a statistically significant participant-by-condition interaction (Table 2; Fig. 2B). Analysis without adjustment for baseline values did not change this inference (participant-by-condition interaction, *p* = 0.02).

At the group mean level, the 2-h change in lactate concentrations was higher with breakfast compared with fasting (*p* < 0.01, Table 2). A large positive correlation of 0.68 (90%CI: 0.24 to 0.89, *p* = 0.01, Fig. 1C) was observed between the 2 replicates of fasting-adjusted 2-h postprandial lactate responses. However, the within-trial SD for lactate was not substantially greater during the breakfast trial than the fasting trial, with both estimates of the individual differences SD below the MCID of ± 0.50 mmol/L and a trivial the participant-by-condition interaction (Table 2; Fig. 2C). Analysis without adjustment for baseline values did not change this inference (participant-by-condition interaction, *p* = 0.28).

At the group mean level, the 2-h change in NEFA concentrations showed a greater decline following breakfast than with extended fasting (*p* < 0.01; Table 2). A moderate positive correlation of 0.48 (90%CI: -0.05 to 0.80, *p* = 0.07, Fig. 1D) was observed between the 2 replicates of fasting-adjusted 2-h postprandial NEFA responses. Both estimates of the individual differences SD were below the MCID of ± 0.29 mmol/L, with a trivial effect for the participant-by-condition interaction (Table 2; Fig. 2D). Analysis without adjustment for baseline values did not change this inference (participant-by-condition interaction, *p* = 0.45).

### Energy expenditure and substrate oxidation

**Fig. 3:**
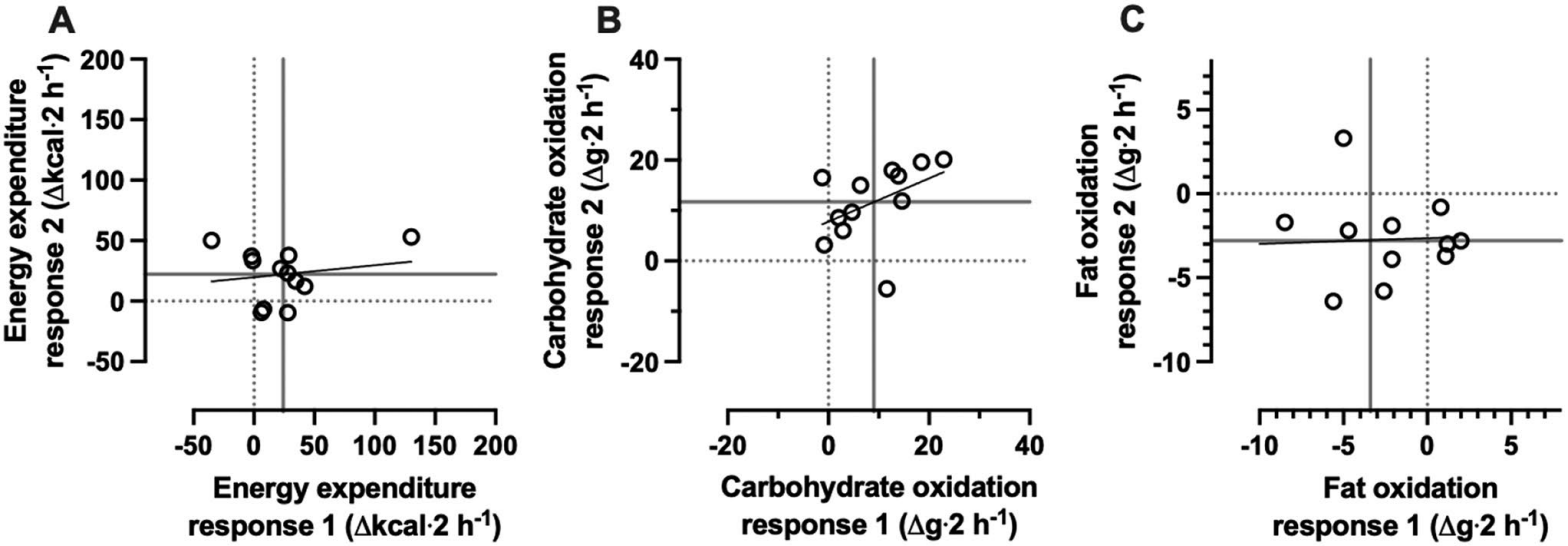
Correlation between the replicates of the baseline-to-two-h response to breakfast minus the fasting control condition, for whole-body energy expenditure (**A**), carbohydrate oxidation (**B**), and fat oxidation (**C**). “Response 1” corresponds to the first pair of conditions (breakfast 1 minus fasting 1) and “Response 2” to the second pair of conditions (breakfast 2 minus fasting 2). Each data point is an individual participant. The dotted lines represent the MCID and the solid lines represent the group mean. *n* = 12. MCID, minimal clinically important difference

**Fig. 4:**
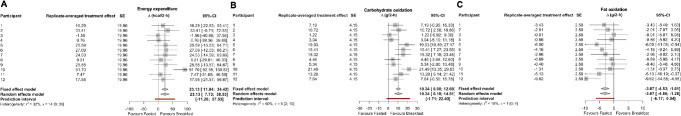
Results of the meta-analysis of each participants treatment effect estimate for whole-body energy expenditure (**A**), carbohydrate oxidation (**B**), and fat oxidation rates (**C**) two h after consumption of breakfast (BREAKFAST) relative to two h after remaining in the overnight fasted state (FASTED). *n* = 12

Whole-body energy expenditure and carbohydrate oxidation were both higher following breakfast consumption than extended overnight fasting (Table 1, *p* < 0.01 and < 0.0001, respectively), whereas fat oxidation was lower with breakfast consumption than extended overnight fasting (*p* < 0.01; Table 2). A small positive correlation was observed between the 2 replicates of fasting-adjusted 2-h postprandial energy expenditure (*r* = 0.18, 90%CI: -0.35 to 0.62, *p* = 0.29; Fig. 3A) moderate positive correlation for carbohydrate oxidation (*r* = 0.44, 90%CI: -0.08 to 0.77, *p* = 0.08, Fig. 3B) and negligible correlation for fat oxidation responses (*r* = 0.07, 90%CI: -0.45 to 0.55, *p* = 0.42, Fig. 3C). The within-trial SD for energy expenditure, carbohydrate and fat oxidation rates were not substantially greater during the breakfast trial than the fasting trial, with trivial effects for the participant-by-condition interaction terms (Table 2; Fig. 4A, B and C).

### Appetite ratings

The 2-h change in ratings of hunger and prospective consumption were both lower following breakfast consumption than extended overnight fasting (*p* < 0.01 and < 0.001, respectively; Table 2), whereas ratings of fullness and satisfaction were both higher following breakfast consumption than extended overnight fasting (*p* < 0.001 and < 0.01, respectively; Table 2).

**Fig. 5:**
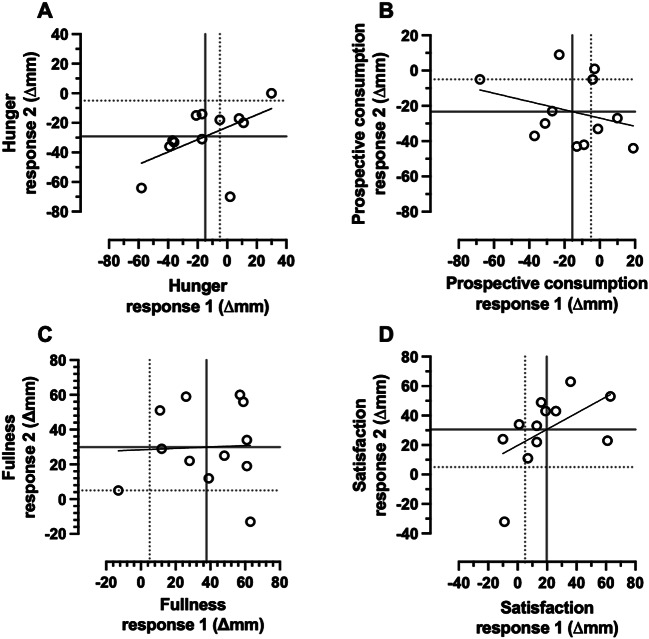
Correlation between the replicates of the baseline-to-two-h response to breakfast minus the fasting control condition, for hunger (**A**), prospective consumption (**B**), fullness (**C**), and satisfaction ratings. “Response 1” corresponds to the first pair of conditions (breakfast 1 minus fasting 1) and “Response 2” to the second pair of conditions (breakfast 2 minus fasting 2). Each data point is an individual participant. The dotted lines represent the MCID and the solid lines represent the group mean. *n* = 12. MCID, minimal clinically important difference

**Fig. 6:**
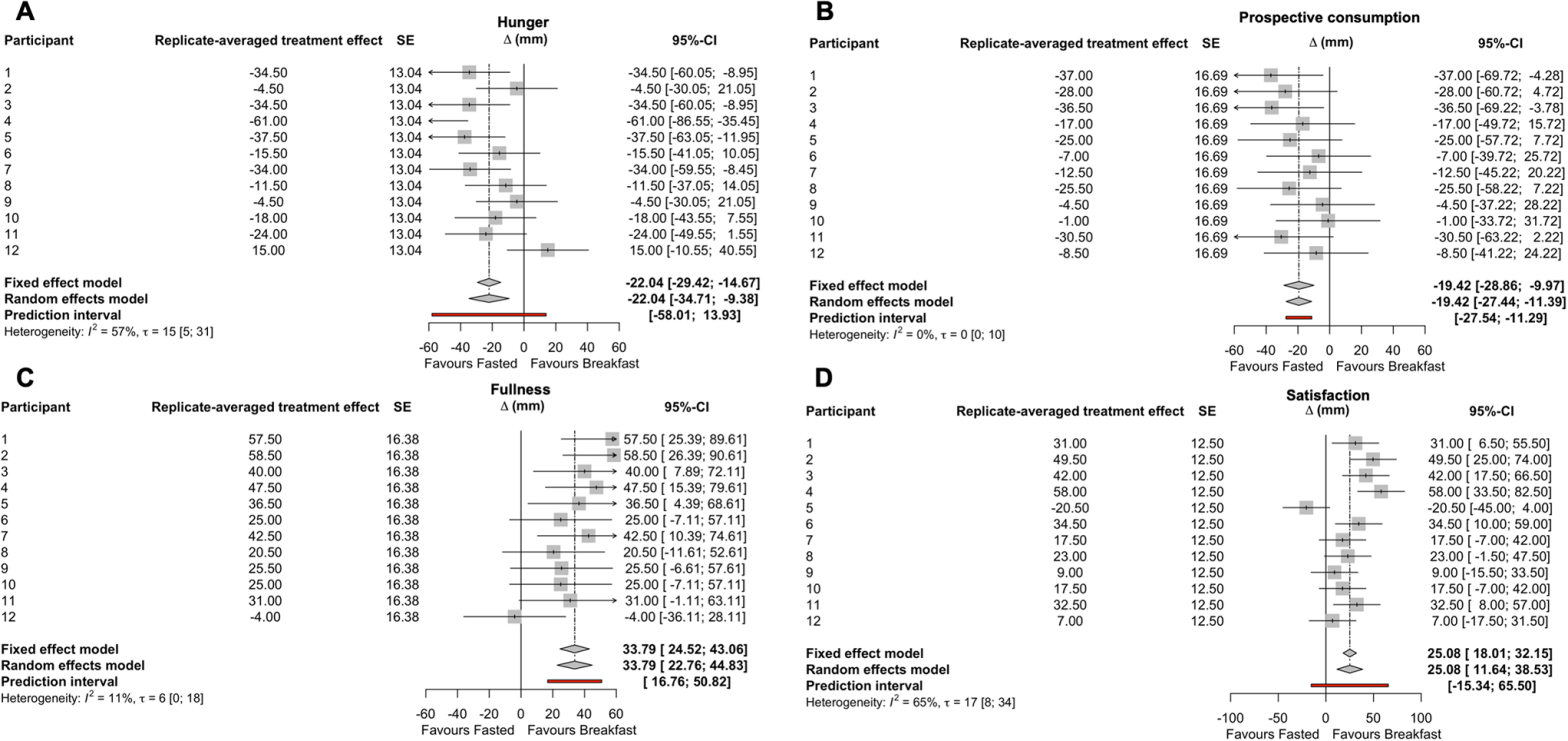
Results of the meta-analysis of each participants treatment effect estimate for hunger (**A**), prospective consumption (**B**), fullness (**C**), and satisfaction ratings (**D**) two h after consumption of breakfast (BREAKFAST) relative to two h after remaining in the overnight fasted state (FASTED). *n* = 12

A large positive correlation was observed between the 2 replicates of fasting-adjusted 2-h change in hunger (*r* = 0.52, 90%CI: 0.03 to 0.81, *p* = 0.04; Fig. 5A). The within-trial SD for hunger was not substantially greater during the breakfast trial than the fasting trial, with a trivial effect for the participant-by-condition interaction (Table 2; Fig. 6A).

A small negative positive correlation was observed between the 2 replicates of fasting-adjusted 2-h change in prospective consumption (*r* = -0.29, 90%CI: -0.69 to 0.24, *p* = 0.18; Fig. 5B). The within-trial SD for hunger was not substantially greater during the breakfast trial than the fasting trial, with a trivial effect for the participant-by-condition interaction (Table 2; Fig. 6B).

A negligible correlation was observed between the 2 replicates of fasting-adjusted 2-h change in fullness (*r* = 0.04, 90%CI: -0.47 to 0.53, *p* = 0.45; Fig. 5C). The within-trial SD for fullness was not substantially greater during the breakfast trial than the fasting trial, and the *p* value for the participant-by-condition interaction was above the threshold for statistical significance (Table 2; Fig. 6C).

A large positive correlation was observed between the 2 replicates of fasting-adjusted 2-h change in satisfaction (*r* = 0.53, 90%CI: 0.04 to 0.81, *p* = 0.04; Fig. 5D). The within-trial SD for hunger was not substantial greater during the breakfast trial than the fasting trial, and the *p* value for the participant-by-condition interaction was above the threshold for statistical significance (Table 2; Fig. 6D).

### Correlations between glucose responses, participant characteristics and other outcomes

Moderate, positive correlations were observed between the pooled, fasting-adjusted glucose response and the pooled, fasting-adjusted insulin (*r* = 0.47, 95%CI: -0.18 to 0.83, *p* = 0.15), lactate (*r* = 0.32, 95%CI: -0.34 to 0.77, *p* = 0.33) and NEFA responses (*r* = 0.40, 95%CI: -0.27 to 0.80, *p* = 0.23). A small, positive correlation was observed between the pooled, fasting-adjusted glucose response and the pooled, fasting-adjusted fullness response (*r* = 0.25, 95%CI: -0.41 to 0.74, *p* = 0.46), whereas correlations between the pooled, fasting-adjusted glucose response and the pooled, fasting-adjusted hunger (*r* = -0.07, 95%CI: -0.64 to 0.56, *p* = 0.85), satisfaction (*r* = 0.06, 95%CI: -0.56 to 0.63, *p* = 0.87), and prospective consumption responses (*r* = -0.16, 95%CI: -0.69 to 0.48, *p* = 0.63) were small-to-negligible. A large, negative correlation was observed between body mass and the pooled, fasting-adjusted lactate response (*r* = -0.59, 95%CI: -0.88 to 0.01, *p* = 0.06), whereas the correlation between body mass and the pooled, fasting-adjusted insulin response was moderate and negative (*r* = -0.37, 95%CI: -0.80 to 0.29, *p* = 0.26).

## Discussion

The present investigation provides evidence of reactive hypoglycaemia 2 h following breakfast consumption at the group (mean response) level, but with undetectable meaningful inter-individual variability. The only postprandial outcome to display meaningful inter-individual variability in response to breakfast consumption was serum insulin concentrations. These data, therefore, do not support the concept that people respond differently to one another with respect to blood glucose “dips” after a breakfast meal, nor that this translates into individual differences in appetite responses. Rather, that individual differences in the insulin response to the breakfast regulate blood glucose concentrations within a range.

Reactive hypoglycaemia in the postprandial state (sometimes referred to as glucose dips) typically occurs 2–3 h after a meal and is thought to be primarily due to insulin-stimulated peripheral glucose uptake superseding the increase in exogenous (meal-derived) glucose appearance rates [35]. Whilst sampling from compartments that drain insulin sensitive tissues (e.g., mixed or deep venous blood, and interstitial fluid) is likely to over diagnose reactive hypoglycaemia compared to arterial, capillary or arterialised blood [36], reactive hypoglycaemia is still observed with arterialised sampling [35] and the phenomenon is therefore not simply an artefact of blood sampling site. It has been suggested that reactive hypoglycaemia displays interindividual variability and contributes to appetite regulation on the basis of correlations between and within individuals [4]. The current study is the first to employ a replicate crossover design with postprandial glucose sampling over a timeframe relevant for capturing reactive hypoglycaemia. The replicate crossover design allows for quantification of the participant-by-condition interaction and thus allows inferences to be drawn about true inter-individual heterogeneity of responses to an intervention [7]. The data in the current study do no provide evidence of meaningful inter-individual heterogeneity of the 2-h glucose response to breakfast consumption when accounting for the control condition of extended overnight fasting. Nor do the data provide evidence for meaningful inter-individual heterogeneity for 2-h lactate or NEFA concentrations, energy expenditure, substrate metabolism, or appetite ratings. The only outcome to show meaningful inter-individual heterogeneity of response to breakfast consumption was serum insulin concentrations.

Insulin is the primary hormone controlling the shift in substrate metabolism from the fasted to the postprandial state. Insulin controls glucose concentrations primarily by suppressing endogenous glucose production and stimulating peripheral tissue glucose uptake. The availability of circulating insulin is dependent on insulin secretion and hepatic insulin extraction. The former of which is stimulated by circulating glucose concentrations and potentiated by the incretin hormones. When the size and composition of the meal is fixed across participants, as is the case in the current study, then the relative macronutrient load differs. Smaller individuals will receive a larger relative proportion of nutrients and thus, all else being equal, would receive a larger signal for insulin secretion. Consistent with this, we observed a negative relationship between body mass and the 2-h insulin response adjusted for the fasting condition. Consequently, it is likely that the interindividual heterogeneity in the insulin response to breakfast resulted in regulatory control of glucose concentrations, such that meaningful interindividual heterogeneity for glucose concentrations were not observed. This principle can also be demonstrated by observations that within-individuals, doubling energy intake increases postprandial insulin concentrations without significantly increasing postprandial glucose concentrations [37].

The postprandial circulating lactate concentrations in response to a meal are the net result of changes in the uptake and release of lactate from tissues. Insulin-stimulated glycolysis can increase lactate production in splanchnic and peripheral tissues [38–40], and the inclusion of specific monosaccharides within a meal, such as fructose and galactose, may further increase lactate concentrations due to their interconversion by the liver and splanchnic bed [32, 41–43]. Accordingly, it might be expected that lactate concentrations display inter-individual variability in response to a fixed size meal due to differences in insulin-stimulated peripheral glycolysis and relative availability of lactate precursors. Whilst we observed a large positive correlation between the fasting-adjusted first and second lactate responses to breakfast consumption, the response variance estimates did not exceed the pre-defined target MCID, with a trivial effect for the participant-by-condition interaction. This may be explained by consistent within-individual responses but relatively low absolute concentrations and between-individual heterogeneity, combined with a relatively small sample size. Since the majority of the hydrolysed carbohydrate in the meal would be glucose (~ 52 g glucose, ~ 9 g galactose and ~ 6 g fructose), the increase in blood lactate concentrations at 2 h is likely to be primarily due to stimulation of glycolysis [38] rather than hepatic interconversion of metabolites. If the galactose and/or fructose content of the meal was higher, there is likely to have been higher absolute lactate concentrations [32, 41]. It is therefore possible that a meal high in fructose and galactose containing sugars may produce greater evidence of inter-individual heterogeneity of the lactate response. The current inferences for inter-individual variability of postprandial lactate responses relate to mixed-macronutrient meals with a modest sugar content.

Postprandial increases in carbohydrate metabolism are mirrored by changes in fat metabolism, including decreased circulating NEFA concentrations from inhibition of net adipose tissue lipolysis. Consistent with this, we observed a mean reduction in 2-h NEFA concentrations with breakfast consumption compared with the fasting control. Whilst we observed a moderate positive correlation between the fasting-adjusted first and second NEFA responses to breakfast consumption, the response variance did not exceed the pre-defined target MCID, with a trivial effect for the participant-by-condition interaction. This is possibly due to two reasons. First, like glucose concentrations, the variance in insulin response results in a controlled suppression of lipolysis to achieve a similar NEFA concentration. Second, suppression of adipose tissue lipolysis is highly sensitive to insulin such that in most postprandial situations adipose tissue lipolysis is maximally suppressed [2, 44], thereby resulting a “floor” effect.

Changes in circulating metabolite and hormone concentrations can be both cause and effect of changes in whole-body substrate metabolism. In the postprandial state, whole-body energy expenditure and carbohydrate oxidation is increased, whereas fat oxidation is decreased. Consistent with this we observed mean increases in energy expenditure and carbohydrate oxidation rates and decreases in fat oxidation rates with breakfast consumption compared with fasting. However, we did not find evidence for true interindividual variability in whole-body energy expenditure or substrate metabolism responses to breakfast consumption. It is possible that measurement error is too large to detect such differences with the current sample size, and/or the lack of baseline measure contributes to observing observations of interindividual variance.

Reactive hypoglycaemia has been shown to correlate with appetite ratings and self-reported energy intake [4]. Accordingly, we also examined the interindividual heterogeneity of the appetite responses to a meal. We observed large positive correlations between the fasting-adjusted first and second hunger and satisfaction responses to breakfast consumption, whereas the equivalent correlations for prospective consumption and fullness were negligible or negative. Furthermore, the additional estimate on interindividual response did not provide evidence for meaningful inter-individual heterogeneity of any of the appetite ratings in response to breakfast consumption. To the best of the authors knowledge, only one other study has examined inter-individual heterogeneity of appetite responses to a meal adopting a replicate crossover design [9]. The prior study provided evidence of meaningful inter-individual heterogeneity of appetite ratings 1-h after consumption of a 1200 kcal breakfast (i.e., estimated SDs for all ratings above 10 mm and significant participant-by-condition interaction). The current study provided a breakfast with less than half the energy content, with appetite ratings determined at the 2-h timepoint. It is, therefore, possible that inter-individual heterogeneity of appetite responses to a meal are dependent on the absolute meal size and the timeframe of measurement. To examine whether reactive hypoglycaemia was associated with appetite ratings, we explored correlations between the pooled, fasting-adjusted glucose responses to breakfast, and the pooled, fasting-adjusted responses for each of the appetite ratings. We found no evidence that reactive hypoglycaemia was associated with meaningful increases in appetite.

The present study sample size and the standard absolute portion size of the meal provided constitute aspects that deserve consideration. This designed experiment is limited by the relatively small sample size, which, however, also reflects the onerous nature of the replicate crossover trial per se that requires rigorous standardization, replicated dietary protocols, and different outcome measure assessments [15, 45]. Nevertheless, it is important to highlight that the fundamental nature of a replicate crossover trial, that aims to separate pragmatically sources of random participant-by-condition interaction variance from within-participant trial-to-trial variability via the repeated assessment of the *same* participants, maximizes study costs in a way that reduces the recruitment pool [45]. Accordingly, our analysis outcomes suggested the number of trial replicates was suitable enough for a reasonably precise estimation of each primary outcome measure participant-by-condition interaction variance components (Table 2). The ability to detect inter-individual heterogeneity for insulin could be, in part, due to a lower day-to-day variability in insulinaemia, yet the day-to-day variability reported for insulin concentrations (~ 0.7%) is only marginally lower than that of glucose (1.1%), lactate (1.5%) or NEFA (1.1%) [46]. The current study estimates based on meta-analysis and SAS modelling outcomes provide the basis to design more replicate crossover studies with differing meal compositions, to assess whether the is low-to-moderate inter-individual heterogeneity of lactate, NEFA and appetite responses to breakfast. Whether any inter-individual heterogeneity of the insulin response to a meal is still evident when meal size is scaled to body size therefore requires further investigation, and requires consideration of how to scale (e.g., for body mass, resting metabolic rate, total energy requirements, fat-free mass, splanchnic and/or skeletal muscle mass). Partly due to these decisions, and also because in practice instant oats are provided in absolute portion sizes, we chose an absolute portion size for the current investigation. This was deemed the most efficient approach, since if there was no evidence for interindividual heterogeneity of response to a fixed size breakfast, then it is unlikely that there would be meaningful interindividual heterogeneity when scaling the breakfast to body size.

To conclude, we were unable to detect any meaningful interindividual heterogeneity of the reactive hypoglycaemia response to breakfast, despite the observation of reactive hypoglycaemia at the group mean level, 2 h after breakfast consumption. We did, however, observe meaningful inter-individual heterogeneity of the 2-h insulin response to breakfast which may partly be explained by differences in body mass and thus relative carbohydrate dose ingested, combined with variance in insulin sensitivity. Whether there is clinically meaningful heterogeneity of the 2-h lactate, NEFA and appetite responses to breakfast require further work, which would benefit from a larger sample size and may depend on the composition of the meal.

## Electronic supplementary material

Below is the link to the electronic supplementary material.

Supplementary Material 1

Supplementary Material 2
