# Supplementary material for: Are there interindividual differences in the reactive hypoglycaemia response to breakfast? A replicate crossover trial
Source: Eur J Nutr. 2024 Sep 4;63(8):2897–909. doi: 10.1007/s00394-024-03467-y (PMC11519142; doi:10.1007/s00394-024-03467-y)
Supplement: Supplementary file 1 — Supplementary Material 1 [file 394_2024_3467_MOESM1_ESM.docx]

**#### glucose ####**

proc mixed data=data covtest ic cl alpha=0.05 nobound plots=residualpanel;

class period treat subject;

model glucose=period treat period*treat glucoseB/ddfm=kr outp=pred cl alpha=0.05 CL vciry residual solution;

random subject subject*XVART;

lsmeans treat /diff cl alpha=0.05;

lsmestimate treat "Breakfast versus Fasted" 1 -1 -1 1/ cl alpha=0.05;

run;

**#### insulin ####**

proc mixed data=data covtest ic cl alpha=0.05 nobound plots=residualpanel;

class period treat subject;

model insulin=period treat period*treat insulinB/ddfm=kr outp=pred cl alpha=0.05 CL vciry residual solution;

random subject subject*XVART;

lsmeans treat /diff cl alpha=0.05;

lsmestimate treat " Breakfast versus Fasted " 1 -1 -1 1/ cl alpha=0.05;

run;

**#### lactate ####**

proc mixed data=data covtest ic cl alpha=0.05 nobound plots=residualpanel;

class period treat subject;

model lactate=period treat period*treat lactateB/ddfm=kr outp=pred cl alpha=0.05 CL vciry residual solution;

random subject subject*XVART;

lsmeans treat /diff cl alpha=0.05;

lsmestimate treat " Breakfast versus Fasted" 1 -1 -1 1/ cl alpha=0.05;

run;

**#### nefa ####**

proc mixed data=data covtest ic cl alpha=0.05 nobound plots=residualpanel;

class period treat subject;

model nefa=period treat period*treat nefaB/ddfm=kr outp=pred cl alpha=0.05 CL vciry residual solution;

random subject subject*XVART;

lsmeans treat /diff cl alpha=0.05;

lsmestimate treat " Breakfast versus Fasted " 1 -1 -1 1/ cl alpha=0.05;

run;

**#### energy expenditure ####**

proc mixed data=data covtest ic cl alpha=0.05 nobound plots=residualpanel;

class period treat subject;

model energyexp=period treat period*treat/ddfm=kr outp=pred cl alpha=0.05 CL vciry residual solution;

random subject subject*XVART;

lsmeans treat /diff cl alpha=0.05;

lsmestimate treat " Breakfast versus Fasted " 1 -1 -1 1/ cl alpha=0.05;

run;

**#### fat oxidation ####**

proc mixed data=data covtest ic cl alpha=0.05 nobound plots=residualpanel;

class period treat subject;

model fatox=period treat period*treat/ddfm=kr outp=pred cl alpha=0.05 CL vciry residual solution;

random subject subject*XVART;

lsmeans treat /diff cl alpha=0.05;

lsmestimate treat " Breakfast versus Fasted " 1 -1 -1 1/ cl alpha=0.05;

run;

**#### carbohydrate oxidation ####**

proc mixed data=data covtest ic cl alpha=0.05 nobound plots=residualpanel;

class period treat subject;

model chox=period treat period*treat/ddfm=kr outp=pred cl alpha=0.05 CL vciry residual solution;

random subject subject*XVART;

lsmeans treat /diff cl alpha=0.05;

lsmestimate treat " Breakfast versus Fasted " 1 -1 -1 1/ cl alpha=0.05;

run;

**#### hunger ####**

proc mixed data=data covtest ic cl alpha=0.05 nobound plots=residualpanel;

class period treat subject;

model hunger =period treat period*treat hungerB/ddfm=kr outp=pred cl alpha=0.05 CL vciry residual solution;

random subject subject*XVART;

lsmeans treat /diff cl alpha=0.05;

lsmestimate treat "Breakfast versus Fasted" 1 -1 -1 1/ cl alpha=0.05;

run;

**#### fullness ####**

proc mixed data=data covtest ic cl alpha=0.05 nobound plots=residualpanel;

class period treat subject;

model fullness=period treat period*treat fullnessB/ddfm=kr outp=pred cl alpha=0.05 CL vciry residual solution;

random subject subject*XVART;

lsmeans treat /diff cl alpha=0.05;

lsmestimate treat "Breakfast versus Fasted" 1 -1 -1 1/ cl alpha=0.05;

run;

**#### satisfaction ####**

proc mixed data=data covtest ic cl alpha=0.05 nobound plots=residualpanel;

class period treat subject;

model satisfaction=period treat period*treat satisfactionB/ddfm=kr outp=pred cl alpha=0.05 CL vciry residual solution;

random subject subject*XVART;

lsmeans treat /diff cl alpha=0.05;

lsmestimate treat "Breakfast versus Fasted" 1 -1 -1 1/ cl alpha=0.05;

run;

**#### consumption ####**

proc mixed data=data covtest ic cl alpha=0.05 nobound plots=residualpanel;

class period treat subject;

model consumption=period treat period*treat consumptionB/ddfm=kr outp=pred cl alpha=0.05 CL vciry residual solution;

random subject subject*XVART;

lsmeans treat /diff cl alpha=0.05;

lsmestimate treat "Breakfast versus Fasted" 1 -1 -1 1/ cl alpha=0.05;

run;
